# Molecular Characterization and Immunological Evaluation of Truncated *Babesia microti* Rhoptry Neck Protein 2 as a Vaccine Candidate

**DOI:** 10.3389/fimmu.2021.616343

**Published:** 2021-02-24

**Authors:** Yu chun Cai, Chun li Yang, Wei Hu, Peng Song, Bin Xu, Yan Lu, Lin Ai, Yan hong Chu, Mu xin Chen, Jia xu Chen, Shao hong Chen

**Affiliations:** ^1^ National Institute of Parasitic Diseases, Chinese Center for Disease Control and Prevention, Shanghai, China; ^2^ Laboratory of Parasite and Vector Biology, Ministry of Public Health, Shanghai, China; ^3^ WHO Collaborating Centre for Tropical Diseases, National Center for International Research on Tropical Diseases, Ministry of Science and Technology, Shanghai, China; ^4^ Department of Clinical Research, The 903rd Hospital of PLA, Hangzhou, China; ^5^ School of Life Sciences, Fudan University, Shanghai, China

**Keywords:** babesiosis, *Babesia microti*, invasion, rhoptry neck protein 2, host immune responses

## Abstract

*Babesia microti* is a protozoan that infects red blood cells. Babesiosis is becoming a new global threat impacting human health. Rhoptry neck proteins (RONs) are proteins located at the neck of the rhoptry and studies indicate that these proteins play an important role in the process of red blood cell invasion. In the present study, we report on the bioinformatic analysis, cloning, and recombinant gene expression of two truncated rhoptry neck proteins 2 (BmRON2), as well as their potential for incorporation in a candidate vaccine for babesiosis. Western blot and immunofluorescence antibody (IFA) assays were performed to detect the presence of specific antibodies against BmRON2 in infected mice and the localization of N-BmRON2 in *B. microti* parasites. *In vitro* experiments were carried out to investigate the role of BmRON2 proteins during the *B. microti* invasion process and *in vivo* experiments to investigate immunoprotection. Homologous sequence alignment and molecular phylogenetic analysis indicated that BmRON2 showed similarities with RON2 proteins of other *Babesia* species. We expressed the truncated N-terminal (33–336 aa, designated rN-BmRON2) and C-terminal (915–1171 aa, designated rC-BmRON2) fragments of the BmRON2 protein, with molecular weights of 70 and 29 kDa, respectively. Western blot assays showed that the native BmRON2 protein is approximately 170 kDa, and that rN-BmRON2 was recognized by serum of mice experimentally infected with *B. microti.* Immunofluorescence analysis indicated that the BmRON2 protein was located at the apical end of merozoites, at the opposite end of the nucleus. *In vitro* red blood cell invasion inhibition studies with *B. microti* rBmRON2 proteins showed that relative invasion rate of rN-BmRON2 and rC-BmRON2 group is 45 and 56%, respectively. Analysis of the host immune response after immunization and *B. microti* infection showed that both rN-BmRON2 and rC-BmRON2 enhanced the immune response, but that rN-BmRON2 conferred better protection than rC–BmRON2. In conclusion, our results indicate that truncated rhoptry neck protein 2, especially its N-terminal fragment (rN-BmRON2), plays an important role in the invasion of host red blood cells, confers immune protection, and shows good potential as a candidate vaccine against babesiosis.

## Introduction

*Babesia microti* is a protozoan parasite that infects red blood cells. It is mainly transmitted by tick bites and can cause fever, anemia and even death in severe cases (1–3). *Babesia* can also be transmitted through blood transfusion. Currently there are no laws requiring screening of blood donors for *Babesia*, so there is a risk of transmission through blood transfusion in China (4, 5).

As in other apicomplexan protozoa, red blood cell invasion by *Babesia* is a complex multistep process, including initial attachment to the host cell, which is a reversible process, followed by parasite realignment. Irreversible attachment and formation of tight junctions with host red blood cells occur rapidly, followed by entry mediated by actin and myosin (6). During the invasion process, parasite proteins and lipids are released from organelles such as the micronemes, rhoptries, and dense granules, located at the apical end of the parasite (7). The subcellular structure of *Babesia* is similar to that of other apicomplexan parasites. The rhoptry is the most important organelle during the invasion process and is comprised of a posterior electron-dense bulb and an anterior electron-lucent neck (8–14). The rhoptry-associated protein (RAP) complex is localized in the rhoptry bulb. Members of this complex include RAP1 and either RAP2 or RAP3 in *P. falciparum* (15). RAP2 and RAP3 are paralogs derived from a gene duplication event in a *P. falciparum* ancestor (16). RAPs have been found in many *Babesia* spp., including *B. bovis, B. bigemina, B. divergence, B. canis, B.motasi-like, B. orientalis*, and *B. gibsoni* (17–22). In total, they have been reported in 12 species, including five RAP genes in *B. motasi*, four RAP genes in *B. bigemina*, four RAP genes in *B. divergence*, two RAP genes in *B. bovis*, and one RAP gene in *B.canis* and *B. microti* (23–28). Rhoptry neck proteins (RONs) receive that name due to their localization at the neck of the rhoptry. These proteins are involved in host cell adhesion and formation of the tight junction between the invading parasite and erythrocyte (29). RONs have been found in many apicomplexan parasites, including *P. falciparum* (e.g., Rh1, 2a, 2b, 4, and 5), and serve as adhesins that bind to host cell receptors (30–36). PfRON2, PfRON4, and PfRON5, in combination with secreted microneme proteins, participate in the formation of the tight junction (37–40). Four RONs in *T. gondii* (TgRON2, TgRON4, TgRON5 and TgRON8) localize at the tight junction during invasion and bind to a microneme protein, *T. gondii* apical merozoite antigen 1 (TgAMA1) (37, 41–44). Prior to 2010 almost no rhoptry neck proteins had been identified (with the exception of those from *Plasmodium* and *T. gondii*), and only some hypothetical proteins had been found in other apicomplexan protozoa. In recent years, however, increasing numbers of *Babesia* rhoptry neck proteins have been reported. For example, Ngabu malobi et al. discovered the rhoptry neck 2 BoRON2 protein in *B. orientalis*. BoRON2 is a polypeptide of 1,345 amino acids (~150 kDa) encoded by a 4,035 bp full-length open reading frame without introns (45). Rosalynn L. Ord et al. confirmed the existence of *B. divergens* and *B. microti* rhoptry neck proteins (BdRON2 and BmRON2, respectively), and proved that they are required for host cell invasion (46). As in other apicomplexan protozoa, *Babesia* rhoptry neck proteins associate with the microneme apical membrane antigen AMA1 to form a tight junction and play an essential role during red blood cell invasion (47).

Cytokines (CK) regulate immune and inflammatory responses. They play an important role in the development and prognosis of babesiosis. According to the different cytokines secreted by CD4+ Th cells, immune responses can be divided into several categories (48), including Th1-type and Th2-type. Th1-type cytokines mainly include interleukin-12 (IL-12), IFN-γ and tumor necrosis factor (TNF-α), whereas Th2-type cytokines mainly include IL-4, IL-5, IL-10 and IL-13. Th1-type cytokines enhance cellular immunity, whereas Th2-type cytokines promote humoral immunity. Hence, IL-12 induces a Th1-type immune response, whereas IL-4 induces a Th2-type immune response. On the other hand, IL-4 and IL-10 inhibit Th1-type immune responses, while IFN-γ inhibits Th2-type immune responses. In many diseases, the proportion of cytokines promoting cellular or humoral immune responses is dysregulated, so cytokines have become one of the research hotspots in babesiosis. In this study we investigated the structure, function and immunological properties of *Bm*RON2, laying the foundation for future investigations.

## Materials and Methods

### Animals and *B. microti* Strain

BALB/c mice were purchased from the Institute of Zoology, Chinese Academy of Sciences, and maintained at the animal care center of the National Institute of Parasitic Diseases, China CDC (IPD, China CDC). The *B. microti* Peabody strain (ATCC, PRA-99) was obtained from the American Type Culture Collection (Manassas, VA) and maintained in BALB/c mice by serial passage, following the method described previously (49).

### Ethics Approval and Consent to Participate

This study was conducted following the guidelines of the Laboratory Animal Welfare & Ethics Committee (LAWEC) of the National Institute of Parasitic Diseases of China CDC (IPD, China CDC) and carried out in strict accordance with the recommendations for the care and use of laboratory animals. Permit Number: IPD-2019-14.

### Sequence Analysis of Rhoptry Neck Protein of *B. microti* (BmRON2)

The EMBL-EBI (http://www.ebi.ac.uk/) and Piroplasma DB (http://piroplasmadb.org/piro/) websites were used to download the BmRON2 gene sequence. ExPASy-PortParam (http://web.expasy.org/protparam/) was used to obtain basic physicochemical information, such as molecular weight, isoelectric point, amino acid composition, atomic ratio, and extinction coefficient of the target protein. ExPASy-ProtScale (http://Web.expasy.org/protscale/) was employed to analyze the hydrophobicity of the protein. TMHMM (http://www.cbs.dtu.dk/services/TMHMM/) and SignalP 4.1 server (http://www.cbs.dtu.dk/services/SignalP/) were employed to analyze the trans-membrane regions and to predict the signal peptide. BmRON2 homologous protein sequences were identified using the Blastp function of the SwissProt database. Next, Clustal-X software was used to analyze amino acid sequence conservation between different species. A phylogenetic tree was constructed using PAUP software and the NJ (Neighbor-Joining) method was used to compare multiple alignments of amino acid sequences.

### Expression and Purification of Recombinant N-BmRON2 and C-BmRON2

Primers were designed to amplify the C- and N-terminally truncated (C-BmRON2 and N-BmRON2) BmRON2 fragments, based on the BmRON2 gene sequences deposited in the GenBank database (**Table 1**). Total *B. microti* RNA was extracted using the EZNA total RNA Kit I from OMEGA, following the kit instructions. For the synthesis of complementary DNAs (cDNA), reverse transcription PCR (RT-PCR) was performed to amplify the target gene. Next, the PCR product was cloned into the pET28a vector to obtain the recombinant plasmid. After identification (based on restriction enzyme analysis and sequencing), *E. coli* BL21 cells were transformed with the recombinant plasmid and induced with Isopropyl β-D-1-thiogalactopyranoside (IPTG). Sodium dodecyl sulphate-polyacrylamide gel electrophoresis (SDS-PAGE) was carried out to identify the expression products. The recombinant protein was purified with a Ni+ ion affinity chromatography column. The purified protein was aliquoted and stored at −80℃ for further use.

**Table 1:** Primer sequences used in this study.

Name	Sequence (5′-3′)	Restriction sites	Description
N- BmRON2-F	CCCaagcttAGTGGTGCAATTCTTCCCCCCAA	Aagctt (*Hind* III)	Forward primer for N-fragment of Bm RON2
N- BmRON2-R	CCGctcgagTATAAAATTCTCCAACTGTTTA	Ctcgag(*Xho* I)	Reverse primer for N-fragment of Bm RON2
C- BmRON2-F	CCCaagcttAGTGGTGCTTACAAATATCTAAGAATAT	aagctt (*Hind* III)	Forward primer for C-fragment of Bm RON2
C- BmRON2-R	CCGctcgagTGAATGTTGAAATTCAATCT	ctcgag(*Xho* I)	Reverse primer for C-fragment of Bm RON2

### Generation of Anti-rBmRON2 Antibodies

We generated polyclonal antibodies against rN-BmRON2 and rC-BmRON2 proteins following a method described previously (48). Briefly, two female New Zealand rabbits (each weighing 2 kg) were subcutaneously injected four times with 100 µl of rN-BmRON2 or rC-BmRON2 proteins (300 µg) at two-week intervals. Ten days after the last immunization, blood samples were collected and the serum was separated by centrifugation. Polyclonal antibodies were affinity purified using rN-BmRON2 and rC-BmRON2-coupled CNBr-activated Sepharose 4B (GE Corporation, Fairfield, CT). Polyclonal antibody titers were measured by ELISA and samples were stored at −80°C until use.

### Western Blot Analysis of Native BmRON2 and r-BmRON2 Proteins

Western blot analysis was performed using standard methods, as described previously (49). Briefly, proteins extracted from iRBCs at 8 days post-infection and from uninfected RBCs, as well as the purified rN-BmRON2 and rC-BmRON2, were fractioned by SDS-PAGE. Gel-separated proteins were transferred to polyvinylidene fluoride (PVDF) membranes (Millipore), which were incubated with rabbit polyclonal anti- rN-BmRON2 antibody (1:500 dilution), or mouse anti-*B. microti* sera (1:500 dilution). Next, HRP-conjugated goat anti-rabbit IgG (1:2,500 dilution) (Sigma-Aldrich, St. Louis, USA), or HRP-conjugated goat-anti-mouse IgG antibody (1:3,000 dilution) (Sigma-Aldrich, St. Louis, USA) was added, and the protein-antibody complexes were visualized using an Enhanced DAB kit (Tiangen Biotech, Beijing, China), following the manufacturer’s instructions.

### Indirect Immunofluorescent Assay (IFA) to Detect Native BmRON2

Indirect immunofluorescent assays (IFA) were performed to localize the native BmRON2 within *B. microti* parasites, as described previously (49). Briefly, infected red blood cells were fixed with 100% methanol on microscope slides. Samples were resuspended in PBS, and then blocked with 5% BSA. After several washes with PBS, the slides were incubated overnight with the rabbit polyclonal anti-rBmRON2 antibody (1:200 dilution). After additional washes, goat anti-rabbit IgG-FITC (488 nm, Santa Cruz Biotechnology, Dallas, TX) (1:2,500 dilution) was added and the samples were incubated for 120 min in the dark. After more washes, the slides were incubated with 6-diamidino-2-phenylindole (DAPI) in the dark for 10 min. Images were examined using a Nikon C2 confocal microscope system and analyzed with NIS-Elements AR software (Nikon, Tokyo, Japan).

### *In Vitro* Inhibition of Invasion of Red Blood Cells by Recombinant BmRON2

Whole blood was collected from *Babesia-*infected BALB/c mice in EDTA-anticoagulant tubes when the parasite density was about 70%, and centrifuged at 1,200 rpm for 10 min. The supernatant, plasma and leukocyte layers were discarded, and the pellet washed twice with Puck’ saline glucose (PSG), and once with PSG plus extra glucose (PSG + G). Infected red blood cells were added to uninfected cells at a ratio of 1:2, so the infection rate of the culture was reduced to 23%. Next, 10 μL of recombinant protein was added to obtain a final concentration of 500, 250, 100, 50, or 10 μg/mL. BSA or 10 μl of HL-20 medium alone (blank control) were added to two parallel cultures as negative controls. The 96-well culture plates (150 μL per well) were placed in a hypoxic cell culture system filled with nitrogen, transferred to a carbon dioxide incubator, and incubated for 48h at 37°C. To calculate the infection rate, ethidium bromide (40 μL of a 20 μg/mL working solution) was mixed with the same volume of culture media and incubated for 30 min at room temperature in the dark, followed by 420 μL of HL20 medium, for a total volume of 500 μL. A total of 50,000 cells were collected and the infection rate was calculated based on the ratio of positive cells versus the total number of cells counted by flow cytometry (50).

The relative invasion rate was calculated as follows:

Relative invasion rate=infection rate of the experimental group/infection rate of the control group

### Immunization With Recombinant BmRON2 Proteins and *B. microti* Infection

BALB/c mice were randomly divided into 3 groups: control group, rN-BmRON2 group, and rC-BmRON2 group. Sera from each group of mice were collected before the initial immunization as a negative control. Recombinant N-BmRON2 and rC-BmRON2 proteins were mixed with Freund’s complete adjuvant and subcutaneously injected on the back of BALB/c mice. Mice were boosted at 14 d, 28 d, and 42 days with antigens emulsified in incomplete Freund’s incomplete adjuvant. Tail blood was collected and the immune titers were measured by ELISA method. Two weeks after the last immunization, 100μL whole mouse blood (20% *B. microti* infection rate) was injected intraperitoneally. Five mice from each group were randomly selected to collect tail blood every other day to measure the infection rate. Five blood samples were collected from the remaining mice and tested on 0d, 7d, 14d, 21d, and 28d post-infection.

### Monitoring Infection in BALB/c Mice

The number of *B. microti*-infected red blood cells per 1,000 was calculated to determine the infection rate on a given day. The weight of the spleen was measured at 0, 7, 14, 21, and 28 days post-infection. Blood cells were analyzed using a Mindray BC-5300 Vet automated hematology analyzer at 0, 7, 14, 21, and 28 days post-infection, following the manufacturer’s instructions. Briefly, whole blood was mixed with the anticoagulant, and “mouse” was selected as the animal type in the blood cell analyzer (version number V01.08.00.16056). The analyzer mode was CBC+DIFF (complete blood count + differential). Eight red blood cell-related markers, 11 white blood cell-related markers and 4 platelet-related markers were measured. The mean plus or minus the standard deviation ($\bar{x}\pm s$) was calculated and statistical analysis was performed with SPSS 21.0 software for each index that exceeded the normal mouse reference range. Differences between two groups were compared using repeated measures analysis of variance. When statistically significant, the LSD method was used for comparisons. Differences were statistically significant when P<0.05.

### Cytokine Quantification With CBA

The Cytometric Beads Array (CBA method) was used to quantify cytokines in infected and control mice. Multiple cytokines in a sample were simultaneously measured using microspheres coated with specific antibodies labelled with different fluorescence markers. The main steps included preparation of cytokine standards, mixed factor capture microspheres, sample incubation, and flow cytometry. After detection, results were collected in FCS2.0 format, and standard curve plots and data analysis were performed using the CBA-specific analysis software FCAP Array (V1.0).

## Results

### Analysis of the BmRON2 Gene

**Figure 1:**
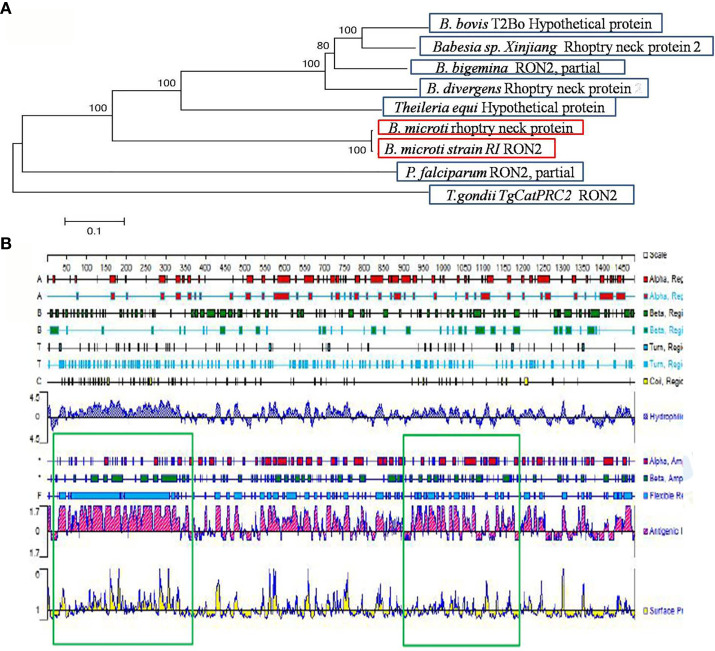
Gene analysis of BmRON2. **(A)** Apicomplexa rhoptry neck protein 2 sequence dendrogram. The dendrogram was constructed using the neighbor-joining method. Bootstrap analysis was performed with 1,000 replicates. **(B)** Signal peptide prediction results for rhoptry neck protein 2. The green boxes show the two truncated protein fragments.

The *B. microti-*related gene (accession number BBM_III04695) found in the Piroplasma DB database and the gene with accession number XP_012649548 found in the EMBL database correspond to the same “rhoptry neck protein gene of *B. microti”*, which is 4, 443 bp long. The BmRON2 protein has a total of 1,480 amino acids, a molecular weight of 165.31 kDa, and an isoelectric point of 8.92. Fragments 468–578aa and 824–1301aa contain two highly conserved structural functional domains. It is a stable and hydrophilic protein, and its N-terminal part is rich in the amino acid methionine. TMHMM analysis showed that the protein has four transmembrane regions: 7–29 aa, 1,256–1,278 aa, 1,377–1,399 aa and 1,414–1,433 aa. The SignalP 4.1 server showed that BmRON2 is a secreted protein with a signal peptide sequence. The BmRON2 amino acid sequence is 99% homologous to *B. microti* strain RI rhoptry neck protein 2, 41% homologous to *Theileria equi* hypothetical protein BEWA_034640, and 36% homologous to *Theileria orientalis* hypothetical protein MACJ_00002836. In addition, it showed 38–39% homology with the following *Babesia* spp. rhoptry-related proteins: hypothetical protein of *B. bovis*, rhoptry neck protein 2 of *B. divergens*, RON2, partial protein of *B. bigemina*, rhoptry neck protein of *B. ovata*, membrane protein of *B. bigemina*, and rhoptry neck protein 2 of *Babesia* sp. *Xinjiang* (**Table 2**). Phylogenetic tree analysis showed that BmRON2 was closely related to other *Babesia* spp. genes and was relatively distant from other species (**Figure 1**).

**Table 2:** Sequence analysis of homologous proteins compared with the RON2s from NCBI.

Species	Gene name	Ident	Accession	Number of amino acids	PUBMED references
*B. microti strain RI*	Rhoptry neck protein 2	99%	XP_021338832.1	1483	27752055/24023759/ 22833609
*Theileria equi*	Hypothetical protein BEWA_034640	41%	XP_004830272.1	1395	23137308
*Theileria orientalis*	Hypothetical protein MACJ_00002836	36%	PVC55603.1	1454	/
*Babesia bovis T2Bo*	Hypothetical protein	39%	XP_001608815.1	1365	/
*Babesia divergens*	Rhoptry neck protein 2	38%	ADM34975.2	1350	/
*Babesia bigemina*	RON2, partial	39%	AQU42588.1	1344	/
*Babesia ovata*	Rhoptry neck protein	39%	GBE61372.1	1344	29078748
*Babesia bigemina*	Membrane protein, putative	39%	XP_012767633.1	1351	/
*Babesia* sp. *Xinjiang*	Rhoptry neck protein 2	39%	ORM40446.1	1012	27784333
*Plasmodium falciparum*	Rhoptry neck protein 2, partial	31%	BAH22613.1	1369	18952195
*Toxoplasma gondii TgCatPRC2*	Rhoptry neck protein RON2	28%	KYK62796.1	1479	/

### Prokaryotic Expression and Purification of Truncated rBmRON2 Proteins

The signal peptide sequence predicted by the SignalP 4.1 server is 1–30 aa, and the four transmembrane regions of the protein are located at the following sites: 7–29 aa, 1,256–1,278 aa, 1,377–1,399 aa and 1,414–1,433 aa. Therefore, the N-terminal amino acid sequence selected for expression (designated as N-BmRON2) skipped the signal peptide and transmembrane regions and included amino acids 33–336. N-BmRON2 is 304 aa long and the gene sequence has 912 bp, its predicted molecular weight is 32.7 kDa, and its isoelectric point is 4.43. The C-terminal sequence selected for expression (designated as C-BmRON2) includes amino acids 915–1,171, it is 257 aa long and the corresponding gene sequence has 771 bp. Its predicted molecular weight is 29.1 kDa and its isoelectric point is 8.86 (**Figure 1**). Analysis with APE software showed that the amplified C-BmRON2 and N-BmRON2 genes were identical to the original gene sequences, but that N-BmRON2 contained one base mutation (the 547th base was changed from A to G).

**Figure 2:**
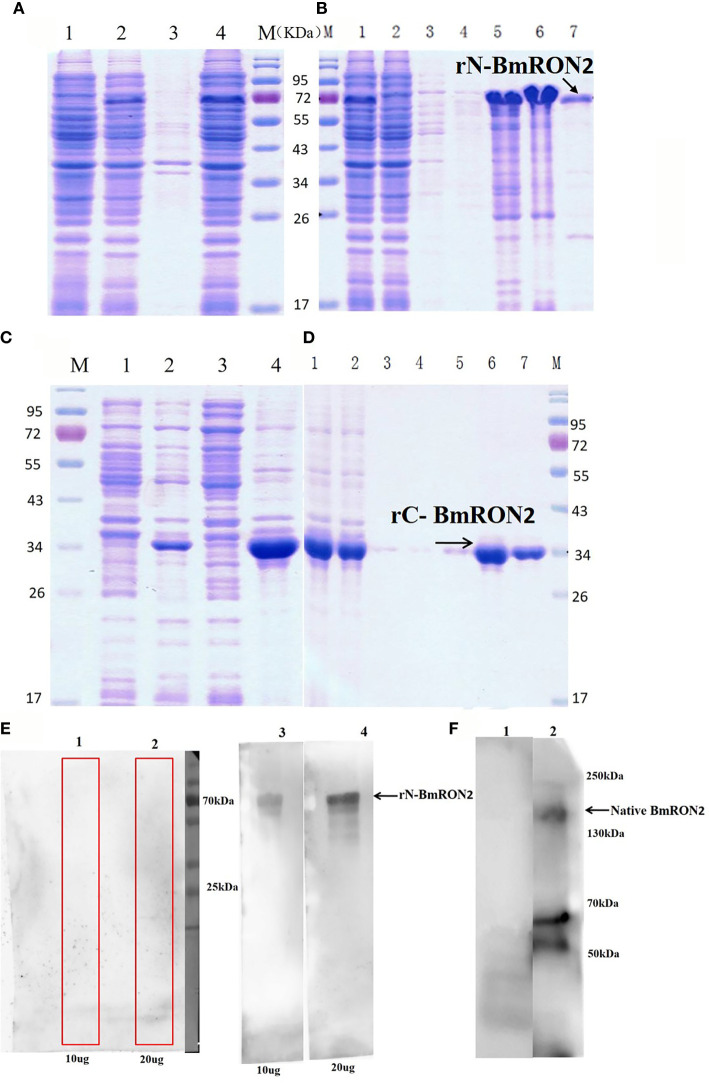
SDS-PAGE and Western blot analysis of native and recombinant BmRON2 proteins. **(A)** (N- BmRON2). M, Protein markers; A1, Uninduced cells, A2, IPTG-induced cells, A3, Supernatant, A 4, Precipitate. **(B)** (N- BmRON2). M, Protein markers; B1, Pre-purified protein, B2, Flow through, B 3-7, Different concentrations of purified protein. **(C)** (C- BmRON2). M, Protein markers; C1, Uninduced cells, C2, IPTG-induced cells, C3, Supernatant, C 4, Precipitate. **(D)** (C- BmRON2). M, Protein markers; D1, Pre-purified protein, D2, Flow through, D 3-7, Different concentrations of purified protein. **(E)** Western blot analysis of rN- BmRON2. E1-2, rN-BmRON2 protein tested with normal mouse serum; E3-4, rN-BmRON2 protein tested with serum from a *B. microti*-infected mouse. **(F)** Western blot analysis of native BmRON2. F1, Uninfected RBCs protein extract tested with rabbit polyclonal anti- rN-BmRON2 antibody, no bands were detected; F2, iRBCs protein extract tested with rabbit polyclonal anti- rN-BmRON2 antibody, a specific band of approximately 170 kDa, plus two additional bands of about 55 kDa to 70 kDa were detected in this sample, suggesting proteolysis of BmRON2.

rN-BmRON2 and rC-BmRON2 were successfully expressed in *E. coli* as His-tagged fusion proteins and purified using Ni-affinity chromatography. SDS-PAGE was performed to evaluate protein expression. Results showed that rN-BmRON2 is a soluble protein that eluted at a concentration of 500 mM imidazole (**Figures 2A, B** **)**. rC-BmRON2 is an inclusion body protein that eluted at an imidazole concentration of 250–500 mM. The predicted molecular weight of rN-BmRON2 is 37 kDa, but after actual expression, its weight was determined to be 70 kDa, which is obviously greater than the theoretical weight. Both recombinant vectors appear to be similar, so this may be related to mutations in this segment of the gene, or to the binding of unknown components which caused the molecular weight of the protein to increase. The specific reason remains unknown and needs to be explored further. In the case of rC-BmRON2, its actual weight (29 kDa) was consistent with the expected MW (**Figures 2C, D** **)**. Western blot analysis indicated that rN- BmRON2 was recognized by serum of mice experimentally infected with *B. microti*, whereas sera from non-infected mice did not recognize it (**Figure 2E**). In contrast, rC-BmRON2 was not recognized by serum of mice infected with *B. microti* (data not shown). These results suggest that N-BmRON2 is highly immunogenic. Western blot assays also showed that a specific protein with a MW of approximately 170 kDa was detected in iRBC lysates prepared on day 8 post-infection, whereas no bands were detected in the uninfected homogenates. The molecular weight of the detected band was in line with the expected size of the full-length BmRON2 protein. However, another two bands of about 55 and 70 kDa were detected in this sample, suggesting proteolysis of BmRON2. These same results were found in the preliminary study (**Figure 2F**).

### Localization of BmRON2 Protein in *B. microti* by Immunofluorescence

**Figure 3:**
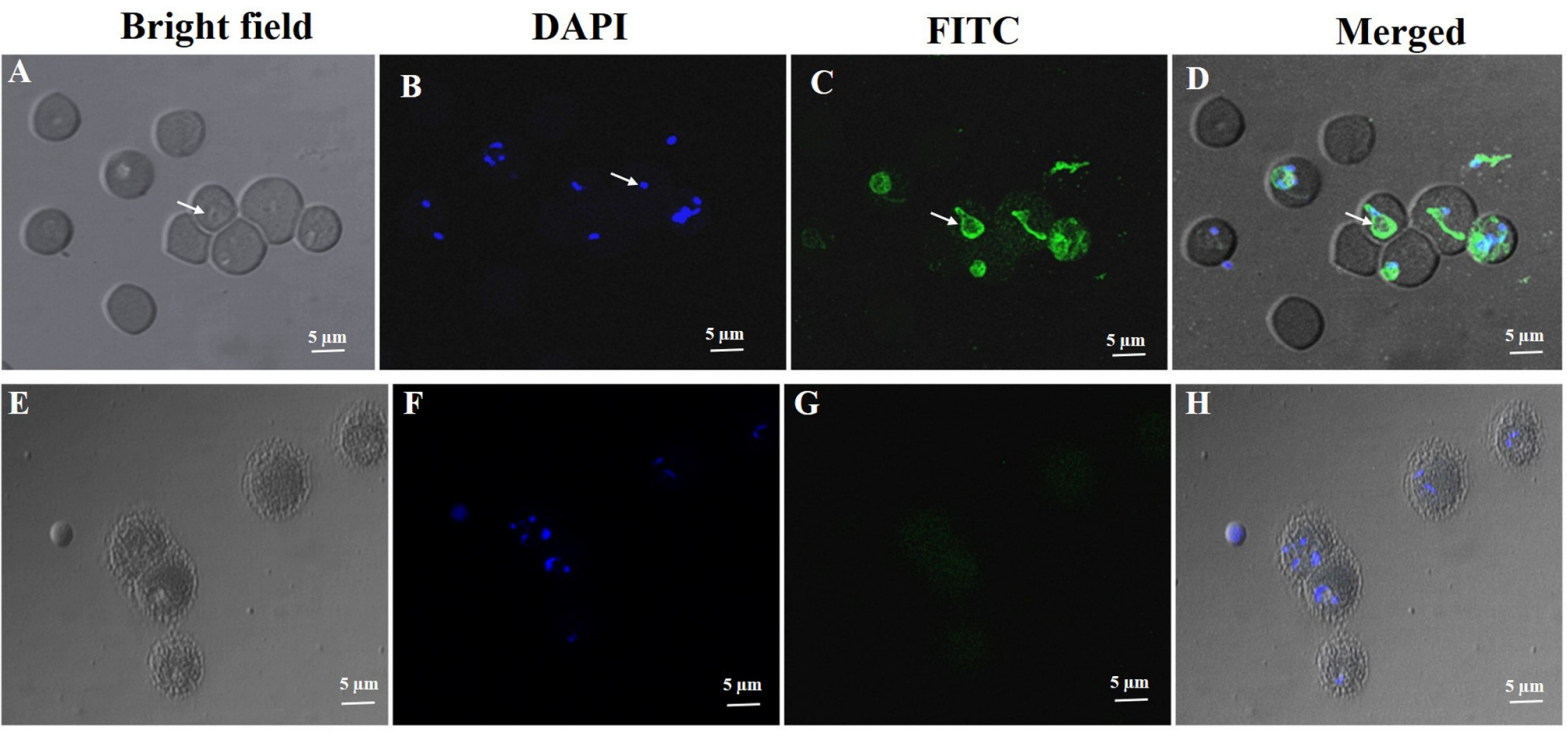
Immunofluorescent localization of BmRON2 in intraerythrocytic *B. microti.* The anti-r BmRON2 antibody reacts with native BmRON2 in the cytoplasm of *B*. *microti*, indicating that BmRON2 is localized at the apical end of the merozoite. White arrows in images **(A–D)** show RBCs infected with *B. microti* parasites. Nuclei are stained blue with 6-diamidino-2-phenylindole (DAPI) **(B)**, and BmRON2 is stained green **(C, D)**, indicating reactivity with the FITC—labelled anti-rabbit IgG and anti-rBmRON2 antibodies. Red blood cells of uninfected mice did not show any specific fluorescent signals **(E–H)**. Scale bar represents 5 µm.

The purified rabbit anti-rN-BmRON2 and anti- rC-BmRON2 antibodies were used to determine the localization of BmRON2 in *B. microti*. Samples were incubated with anti-rN-BmRON2 and anti-rC-BmRON2 antibodies, then with goat anti-rabbit IgG-conjugated to FITC and counterstained with 4′, 6-diamidino-2-phenylindole (DAPI) to stain the nuclei of intraerythrocytic parasites. In the case of C-BmRON2, its localization could not be determined in this study (figure not shown). In the case of N-BmRON2, DAPI stained the parasite nuclei blue (**Figure 3B**), while the antibody labeled the native RON2 and showed green (**Figure 3C**). These results indicate that BmRON2 and the nucleus are located at opposite ends of *B. microti* merozoites, and that BmRON2 is located at the apical end (**Figures 3A–D**). Red blood cells of normal mice did not show any specific fluorescent signals (**Figures 3E–H**).

### Inhibition of RBC Invasion by rN-BmRON2 and rC-BmRON2

**Figure 4:**
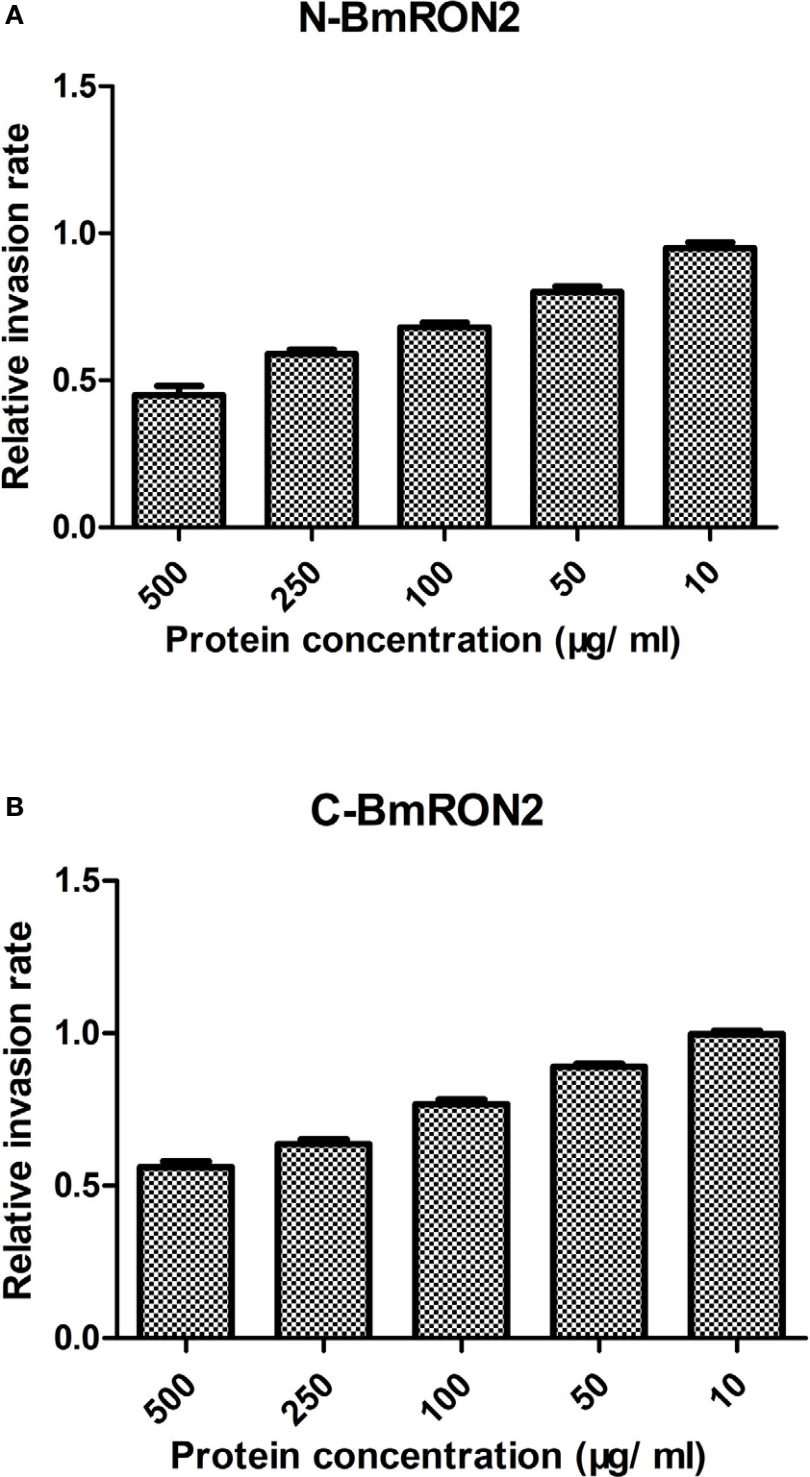
*In vitro* inhibition of red blood cell invasion by *B. microti* rBmRON2 proteins. Inhibitory effects of rN-BmRON2 and rC-BmRON2 on red blood cell invasion. Recombinant proteins were added to the culture medium at concentrations between 10 and 500 μg/mL. rN- BmRON2 **(A)** and rC- BmRON2 **(B)** showed a dose-dependent inhibitory effect.

To investigate the *in vitro* effects of the recombinant proteins on the process of *B. microti* invasion, different concentrations were added to the culture medium. After 48 h of incubation, the infection rates were calculated by flow cytometry, by gating fluorescent cells corresponding to *B. microti*-infected erythrocytes. The infection rates were recorded for the experimental groups and the control group, and the relative infection rates were calculated, as described in the methods section. The results showed that both rN-BmRON2 and rC-BmRON2 inhibited invasion to a certain degree. This inhibitory effect was concentration-dependent and was strongest when the protein concentration was 500 μg/ml. At this concentration, the relative invasion rates in the presence of rN-BmRON2 and rC-BmRON2, were 45 and 56%, respectively. The strongest inhibitory effect was seen with rN-BmRON2. This inhibitory effect was concentration-dependent and was almost undetectable at 10 μg/ml (**Figure 4**).

### *In Vivo* Immune Protective Effect of rBmRON2

**Figure 5:**
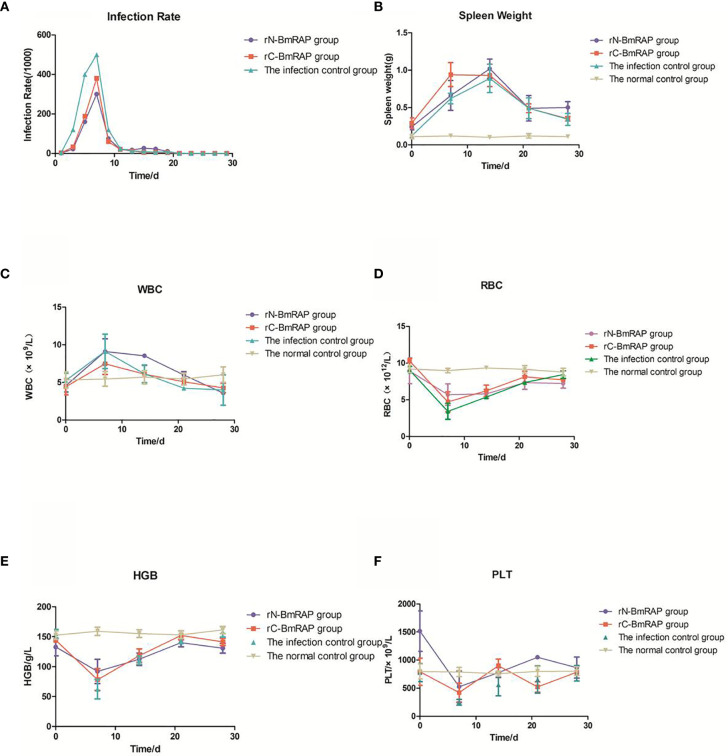
*In vivo* immune protective effect of rBmRON2 proteins in *B. microti* infected mice. **(A)** Infection rates; **(B)** Changes in spleen weight; **(C)** White blood cell (WBC) counts; **(D)** Red blood cells (RBC) numbers; **(E)** Hemoglobin (HGB) content; **(F)** Platelet (PLT) numbers. Normal (uninfected) control group (yellow line, inverted triangles); Infected control group (green line, triangles), rC-BmRON2 group (red line, squares), rN-BmRON2 group (purple line, circles).

After three immunizations with rN-BmRON2 and rC-BmRON2, blood was collected from the mice, and serum was prepared by centrifugation to measure the antibody titers. The average anti-rN-BmRON2 antibody titer produced in BALB/c mice was 8.5×10^4^, whereas the anti-rC-BmRON2 antibody titer was 8.2×10^4^. Both groups of mice produced specific antibodies with high titers, which allowed us to proceed with the *in vivo* infection experiments. Therefore, 18 mice with high titers were selected from each group for subsequent experiments. Analysis of blood smears showed that the infection peaked on the 7th day after infection (the infection rates in the rN-BmRON2, rC-BmRON2, and control groups were 30, 38, and 50%, respectively) (**Figure 5A**). Morphologically, the spleens of the mice were enlarged after immunization with the recombinant proteins and were especially enlarged after infection with *B. microti*. In the infected control group, the spleen showed the largest size at 14 days after infection, reaching a length of 34.33 mm. On the 14th day after infection, the spleen features and size in the three groups reached peak values. The spleen changes in the rN-BmRON2 and rC-BmRON2 groups were less noticeable than in the infected control group, but nonetheless this organ differed significantly when compared with the normal control group (**Figure 5B**). Whole blood cell analysis showed that the number of white blood cells (WBC) peaked on the 7th day after infection, and then decreased. Analysis of variance showed there were no significant differences between the groups (F= 1.842, P>0.05) (**Figure 5C**). The total number of white blood cells, neutrophils, monocytes, lymphocytes, and eosinophils in the two experimental groups and the infected control group fluctuated over time. The number of red blood cells (RBC) and the hemoglobin content (HGB) reached their lowest values on the 7th day after infection. Interestingly, the number of RBCs in the rN-BmRON2 and rC-BmRON2 groups was higher than in the infected control group, but lower than in the normal control group. The RBC numbers gradually recovered with the reduction in the infection rate, and the RBC number in infected mice was close to normal after 21 days. There were significant differences between groups 7 and 14 days after infection (F=8.096, P<0.05) (**Figures 5D, E** **)**. The number of platelets (PLT) reached the lowest value on the 7th day after infection and then gradually recovered. Interestingly, the number of platelets in the rN-BmRON2 and rC-BmRON2 groups was higher than in the infected control group but lower than in the normal control group (**Figure 5F**).

### Analysis of Host Immune Responses After BmRON2 Immunization and *B. microti* Infection

**Figure 6:**
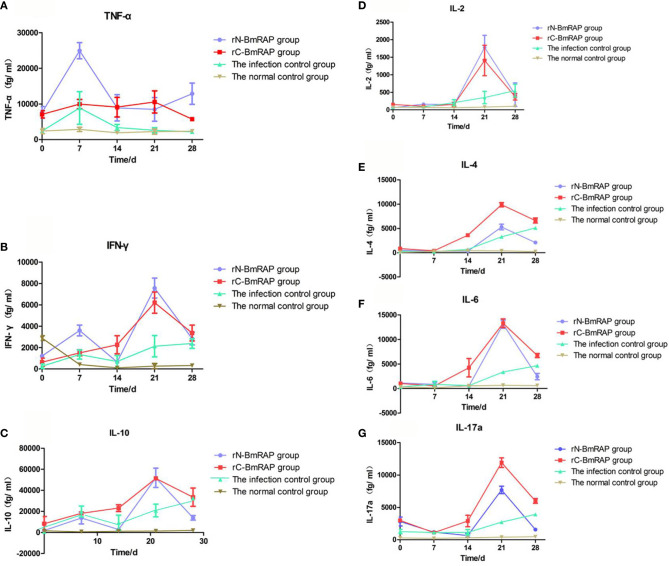
Cytokine concentrations. **(A)** Tumor necrosis factor (TNF-α); **(B)** Interferon-gamma (IFN-γ); **(C)** IL-10; **(D)** IL-2; **(E)** IL-4; **(F)** IL-6; **(G)** IL-17α. Normal control group (brown line, inverted triangles); Infected control group (green line, triangles), rC-BmRON2 group (red line, squares), rN-BmRON2 group (purple line, circles).

TNF-α and IFN-γ levels increased in the infected control group, reaching peak values on the 7th day after infection, and then gradually decreased to pre-infection levels. TNF-α and IFN-γ levels in the rN-BmRON2 and rC-BmRON2 groups were higher than in the infected control group, especially in the rN- BmRON2 group, where this trend was more obvious (**Figures 6A, B** **)**. IL-10 levels in the infected control group peaked on the 7th day after infection, and then decreased slightly, but continued to increase after 14 days of infection. IL-10 levels in the rN-BmRON2 group increased after infection, reaching a peak on the 21st day. The overall IL-10 level trend in the rC-BmRON2 group was similar to what was seen in the infected control group (**Figure 6C**). In the rN-BmRON2 and rC-BmRON2 groups, IL-2, IL-4, IL-6 and IL-17a levels peaked on the 21st day after infection, but then decreased rapidly. In contrast, in the infected control group, IL-2, IL-4, IL-6, and IL-17a levels rose slowly and did not change significantly after day 21 post-infection (**Figures 6D–G**).

## Discussion

The *B. microti*-related gene (Accession No. BBM_III04695) found in the Piroplasma DB database and the gene (Gene Bank accession number XP_012649548) retrieved from the EMBL database are both 4,443 bp long. APE software was used to perform sequence alignment, and the results showed that both gene sequences were identical. However, there are few reports on rhoptry neck proteins related to *B. microti*. The gene investigated in this study was the same rhoptry-associated gene reported by Rosalynn et al. (46), further validating the search method used in this study. Rosalynn et al. identified the RON2 proteins of *B. divergens* (BdRON2) and *B. microti* (BmRON2). This was the first characterization of these proteins in two human babesiosis species, and *B. microti* RON2 was found to have a MW of about 170 kDa. Guanbo Wang et al. (51) also identified these proteins by immunoblotting with mouse anti-rBmRON2 sera and detected distinct bands at 170 and 52 kDa, which is consistent with our results. Homologous sequence alignment and molecular phylogenetic analysis showed that BmRON2 is similar to RON2 proteins of other *Babesia* species and that their phylogenetic relationship is close, which is consistent with other research reports (46). Although the secondary structure of this protein has been predicted, its function and properties are still unknown.

The molecular weight of BmRON2 is relatively high. Since there is no prokaryotic expression method for macromolecular proteins, which may show poor immunospecificity and, in addition, the signal peptide and transmembrane regions are difficult to express, a truncated expression approach was adopted. Guanbo Wang et al. expressed a truncated gene encoding the predicted transmembrane regions 2 and 3 of BmRON2 as a His fusion recombinant protein. The MW of this rBmRON2 was approximately 18 kDa, but it was not recognized by serum from a hamster in a Western blot, and exhibited only limited protection against *B. microti* challenge (51). In our study, gene fragments coding for 33–336aa and 915–1,171aa were expressed as rN-BmRON2 and rC-BmRON2. The recombinant sequences were compared with the original gene sequence using APE software. We found that C-BmRON2 was identical to the original gene sequence, but that N-BmRON2 had one base that did not match the target gene sequence (the 547th base was mutated from A to G). The molecular weight of rN-BmRON2 was predicted to be 37 kDa, but in the actual expressed protein the molecular weight was 70 kDa, which is significantly larger than the theoretical value. According to our experimental results, the recombinant vector is similar, so the larger molecular weight may be related to the mutation, or perhaps the protein binds to unknown components, which causes the molecular weight of the protein to increase. The specific reasons need to be further explored. Western blot assays indicated that rN-BmRON2 was recognized by serum from mice experimentally infected with *B. microti.* Hence, rN-BmRON2 is immunogenic and can protect against *B. microti* infection.

The moving junction is critical for successful penetration of the host cell by apicomplexan parasites. This moving junction, formed by a complex between AMA1 and rhoptry neck protein 2 (RON2), has been well studied in *Plasmodium* and *Toxoplasma*, and is believed to be conserved among apicomplexan parasites. Recent studies identified and characterized the AMA1 and RON2 genes from *B. microti* (46, 52). Guanbo Wang et al. found that combined immunization with rBmAMA1 and rBmRON2 was an effective protective strategy against *B. microti* (51). In our study, the AMA1 protein was not expressed and interactions between AMA1 and RON2 were not investigated. Future studies may provide additional insights.

The RON2 protein is one of the most important *Babesia* molecules involved in red blood cell invasion. It is also one of the most important candidate molecules for inclusion in a vaccine. *Babesia* merozoites bind to red blood cell surface receptors, invade and reproduce inside red blood cells. The proliferation of *Babesia* parasites leads to red blood cell rupture, releasing merozoites. *Babesia* then invades new erythrocytes, triggering a new round of autologous infection (53). The main clinical manifestations in babesiosis patients are a decrease in the number of red blood cells and platelets. Although there are reports in the literature that the red blood cell count, hemoglobin content and other indicators are reduced to varying degrees during *Plasmodium* infections, there are few studies on babesiosis patients. In this study, we monitored the blood indices in detail, and found that the peak reduction in RBC, HGB and PLT values occurred on the 7th day post-infection. The infection rates in the two BmRON2 groups (7 days after infection) were lower than in the infected control group, but N-BmRON2 produced better results than C-BmRON2. However, the mechanisms underlying the protective immune effects of the two truncated BmRON2 proteins need to be explored further.

BALB/c mice are an effective model for studying the mechanism of resistance to *Babesia* (54). The spleen is the largest immune organ in mice, containing large numbers of lymphocytes and macrophages, and is the center of cellular and humoral immunity. Abundant evidence (55) suggests that the spleen plays an important role in the clearance of parasites and parasite-infected red blood cells, but the spleen’s role in parasitic infections and the underlying mechanisms are still unclear. We found that after infection with *B. microti*, the spleen was swollen and its weight increased, indicating that a complex immune response occurred in the mouse body to combat infection. In the infected control group, the spleen length peaked at 14 days after infection, but the spleen changes in the rN-BmRON2 and rC-BmRON2 groups were lower than in the infected control group, indicating that these proteins had a certain protective effect against infection and reduced the degree of stimulation of the inflammatory response. Studies have reported that cellular and humoral immunity play an important role in the fight against intracellular parasitic infections. During the acute infective phase by *B. microti* (56) Th1-type cytokines increase, including IL-12, IFN-γ and TNF-α, and these are essential for controlling the proliferation of parasites (57). In particular, TNF-α (58, 59) is a cytokine that plays an important role in *Babesia* and Plasmodium infections, and can affect the severity of the disease. In this study, we found that TNF-α expression was highest on the 7th day post-infection; that is, when the parasite density peaked, and that TNF-α increased in parallel with the parasite density, which is consistent with previous studies (58, 59), confirming that TNF-α plays an important role during infection. The change in TNF-α concentration in the rN-BmRON2 group was more obvious, indicating that the rN-BmRON2 protein can play an immunoprotective role during *Babesia* infection.

Clawson et al. (60) found that IFN-γ-deficient mice developed mild parasitemia after infection with *B. microti*, but eventually cleared the infection. In contrast, the state of parasitemia in B cell deficient mice did not differ from the control group. These data indicate that cellular immunity is critical for BALB/c mice to resist *Babesia* infection. Buddle et al. (61) hypothesized that IFN-γ is an important component of the Th1-type immune response which mediates resistance to infection. On the other hand, Th2-type cytokines, such as IL-4 and IL-10, have contrasting roles with respect to Th1-type factors. In this study, IL-10 levels in the control group were higher than in the experimental group on the 7th day after infection. Jeong et al. (62) found that IL-10 promoted invasion by *Babesia*, and that parasite density was high when IL-10 expression was high. This is consistent with the results of this study. However, they only measured this cytokine 14-day post-infection. Our study found that IL-10 levels in the experimental groups were higher than in the control group on day 21 after infection. The specific reasons still need to be investigated. In this study we only investigated some immune mechanisms induced by BmRON2, such as cytokine indexes. A more comprehensive evaluation of immune cells and antibodies may better clarify the protective immune mechanisms. Pathological changes or the effects of knocking out the BmRON2 gene may provide additional insights.

## Conclusions

Homologous sequence alignment and molecular phylogenetic analysis showed that BmRON2 is similar to the BmRON2 proteins of other *Babesia* species. A truncated recombinant expression approach was adopted to generate rN-BmRON2 and rC-BmRON2, with molecular weights of 70 and 29 kDa, respectively. The native BmRON2 protein is approximately 170 kDa. rN-BmRON2 was recognized by serum from mice experimentally infected with *B. microti.* The BmRON2 protein and the nucleus are located at opposite ends of *B. microti* merozoites—BmRON2 is located at the apical end. rN-BmRON2 and rC-BmRON2 inhibited red blood cell invasion by *B. microti* to a certain degree (45 and 56%, respectively). *In vivo* analyses of host immune responses after immunization and *B. microti* infection showed that both rN-BmRON2 and rC-BmRON2 enhanced the immune response, but that the immune protection triggered by rN-BmRON2 was better.

## Data Availability Statement

The original contributions presented in the study are included in the article/supplementary material. Further inquiries can be directed to the corresponding authors.

## Ethics Statement

The animal study was reviewed and approved by the Laboratory Animal Welfare & Ethics Committee (LAWEC) of the National Institute of Parasitic Diseases of China CDC (IPD, China CDC).

## Author Contributions

YCC, CLY, and JXC conceived and designed the experiments. CLY, YCC, WH, PS, BX, YL, LA, MXC and YHC performed the experiments. CLY and YCC analyzed the data. YCC, CLY, JXC and SHC wrote the paper. All authors contributed to the article and approved the submitted version.

## Funding

This work was supported by the Fund of the Shanghai Municipal Commission of Health and Family Planning (201940236), the National Parasitic Resources Center, the Ministry of Science and Technology (NPRC-2019-194-30), and the National Key Research and Development Program of China (No. 2016YFC 1202000, 2016YFC 1202005).

## Conflict of Interest

The authors declare that the research was conducted in the absence of any commercial or financial relationships that could be construed as a potential conflict of interest.

## Abbreviations

BmRON2, *Babesia microti* rhoptry neck protein 2; C-BmRON2, C-terminal truncated *Babesia microti* rhoptry neck protein 2; cDNA, complementary DNA; CK, cytokine; ddH2O, double distilled H2O; EDTA, ethylene diamine tetraacetic acid; ELISA, enzyme-linked immunosorbent assay; HRP, horseradish peroxidase; IFN-γ, interferon gamma; IPTG, isopropyl-β-d-thiogalactoside; N-BmRON2, N-terminal truncated *Babesia microti* rhoptry neck protein 2; ORF, open reading frame; PBS, phosphate buffer saline; PCR, polymerase chain reaction; PLT, blood platelet; RBC, red blood cell; SDS-PAGE, sodium dodecyl sulfate-polyacrylamide gel electrophoresis; TNF, tumor necrosis factor; WBC, white blood cell.
